# Wearable devices as part of postoperative early warning score systems: a scoping review

**DOI:** 10.1007/s10877-024-01224-4

**Published:** 2024-10-08

**Authors:** E. G. Bignami, M. Panizzi, F. Bezzi, M. Mion, M. Bagnoli, V. Bellini

**Affiliations:** Anesthesiology, Critical Care and Pain Medicine Division, Department of Medicine and Surgery, University of Parma, Viale Gramsci 14, 43126 Parma, Italy

**Keywords:** Early warning system, IoT, Postoperative care, Postoperative monitoring, Telemedicine, Wearable devices

## Abstract

Postoperative deterioration is often preceded by abnormalities in vital parameters, but limited resources prevent their continuous monitoring in patients with no indication to ICU admission. The development of new technologies allowed the introduction of wearable devices (WDs), enabling the possibility of postoperative monitoring in surgical wards. We performed a Scoping Review to determine the current use of wearable devices as part of Continuous Remote Early Warning Score (CREWS) systems and their efficiency during postoperative period. This Scoping Review was conducted according to PRISMA-ScR guidelines. PICO framework was used before the search to define the review protocol. Systematic literature research has been performed on PubMed, MeSH, MEDLINE and Embase, considering a period between 2018 and February 2024. Prospective and retrospective studies involving patients undergoing cardiac and non-cardiac surgery are included. A total of 10 articles were included in the review. 11 different CE/FDA approved wearable devices were used in the studies analyzed. In all studies the WDs were applied the day of the surgery. The use of WDs as part of CREWS systems is feasible and safe. Furthermore, with the aid of other technologies (LoRa and Artificial Intelligence), they shorten Length of Stay (LOS) and reduce the number of ICU admissions with a reduction in healthcare costs. Continuous monitoring in surgical departments can facilitate the correct and timely identification of postoperative complications. This article is a starting point for the development of new protocols and for the application of these monitoring systems in clinical practice.

**Supplementary Information:**

The online version contains supplementary material available at 10.1007/s10877-024-01224-4.

## Introduction

Surgery represents an invasive and traumatic event for patients. Surgical injury is a major physiological stress factor, triggering a powerful hormonal stress response with the release of mediators including catecholamines, cortisol, and glucagon. The resulting systemic changes can have serious consequences, particularly in vulnerable groups such as the elderly, obese, or those with comorbidities [1–3]. Many complications occur in the early postoperative period and are associated with significant morbidity and mortality, prolonged hospital stay, re-operation, readmission to hospital, and increased costs [4–7]. Numerous studies have shown that deterioration is often preceded by abnormalities in vital signs. Early indicators of clinical deterioration can be detected up to 48 h before an unplanned ICU admission from the surgical ward, especially in high-risk patients and after major procedures, where the risk of complications ranges from 17 to 44% [8]. The need to intercept the onset of adverse events as early as possible in the first postoperative days, a critical period for successful patient outcomes, leads clinicians to consider very carefully the level of postoperative care required for each clinical case. Inevitably, the hospital's available resources become a key factor in this process. The patient's clinical condition, combined with the complexity of the surgical procedure, guides the decision to evaluate the most appropriate level of postoperative monitoring [9].

However, it is well known that in our daily practice we must deal with situations where we have patients who do not meet the criteria for close monitoring in an intensive or sub-intensive care unit, but for whom monitoring in a surgical ward may not be sufficient.

As continuous monitoring of vital parameters is not available in the general medical ward, the nursing staff performs intermittent checks to monitor the patient's vital parameters. During these checks, nurses measure various vital signs, often followed by manual entry and, in some specific cases, calculation of an early warning score, such as the Modified Early Warning Score (MEWS) [10], to identify patients at risk of deterioration. The MEWS is one of the first Early Warning Scores (EWS). Many other variants exist, such as the Modified Early Obstetric Warning Score (MEOWS) or the UK's National Early Warning Score (NEWS), but the purpose for all is to timely detect clinical deterioration to early activate the hospital emergency team. This leads to the reduction of inappropriate ICU admissions or an early ICU admission, reducing the Failed to Rescue rate [11, 12]. In clinical practice, manual checks represent a significant workload, which means that vital signs are often poorly recorded. Additionally, these checks capture only vital parameters at a specific moment, and vital parameters during the rest of the day remain unknown. Many studies have shown that deterioration is often preceded by abnormalities in vital signs [13, 14]. Continuous postoperative monitoring of vital parameters on the ward would therefore be useful because it is well known that the presence of an intensivist staffing model alone is not enough to improve mortality [15, 16].

In response to this need, the development of new technologies has led to the introduction of cutting-edge wearable devices that enable the continuous recording of vital parameters. These devices facilitate postoperative monitoring even for patients whose clinical conditions do not meet the criteria for ICU monitoring but still require more precise observation. These wearable wireless devices are capable of continuously recording and transmitting several vital parameters such as heart rate, respiration rate, saturation, and blood pressure, thereby facilitating remote continuous monitoring of vital signs in general hospital wards [14]. Nowadays, several wearable devices exist for the continuous monitoring of vital parameters that may allow us to move in a direction where the future choice of postoperative monitoring will no longer only be between intensive/sub-intensive departments rather than in general departments, but giving the possibility of sending the patient to a general department with continuous monitoring without overloading nursing work, while simultaneously reducing late intervention in case of adverse events [17].

With this Scoping Review, we will investigate the current use of wearable devices as part of a Continuous Remote Early Warning Score (CREWS) systems in the postoperative period.

## Methods

This Scoping Review was conducted according to the Preferred Reporting Items for Systematic Reviews and Meta-analyses for Scoping Reviews (PRISMA-ScR) guidelines.

PICO Framework was used before the research to define the review protocol (not registered) [Table 1].

**Table 1:** PICO framework

Question type	Patient, population, problem	Intervention or exposure	Comparison or control	Outcome measures
Determine the current use of wearable devices as continuous vital parameters monitors and their efficiency as early warning system during postoperative period	Adult patients undergoing cardiac or non cardiac surgery with programmed or not programmed ICU admission	Adult patients undergoing cardiac or non cardiac major surgery	Standard Early Warning Scores (EWS)	Device’s performance as part of an Early Warning Score (EWS) system and their ease of use

The Author's objective was to determine the current use of wearable devices as continuous vital parameters monitors and in particular their efficiency as part of CREWS systems and their ease of use during the postoperative period. To summarize the results, this type of data was collected: (1) year, (2) country, (3) study design, (3) type of surgery, (4) monitoring setting, (5) objective of the study, (6) number of patients in the final cohort, (7) parameters monitored, (8) duration of the monitoring, (9) summary of the results. A table was compiled for each study included where the results were summarized. We described the devices and protocols in two different sections.

The authors performed a systematic literature search of PubMed, MeSH, MEDLINE, and Embase, between 2018 and 2024 (a 5 year period where technology it is assumed as homogeneous under a technical point of view) using these queries: ((wearables) AND (early warning system)) AND (postoperative), ((wearable) AND (complications)) AND (postoperative), ((wearable) AND (monitoring)) AND (surgery), ((wearables) AND (vital parameters)) AND (postoperative), ((wearable device) AND (blood pressure)) AND (postoperative), ((wearable device) AND (haemodinamics)) AND (postoperative), ((wearable device) AND (saturation)) AND (postoperative), ((wearable device) AND (heart rate)) AND (postoperative); ((postoperative) AND (sweat)) AND (lactate); ((wearables) AND (postoperative)) AND (lactate); ((wearable) AND (lactate)) AND (device).

Both prospective and retrospective studies were eligible for inclusion. The most recent search was made in February 2024. Three authors reviewed and selected the unique original articles independently before reaching consensus on the final set. After the removal of duplicates were considered non-eligible non-English articles, non-original articles. No automated tools were used.

**Fig. 1:**
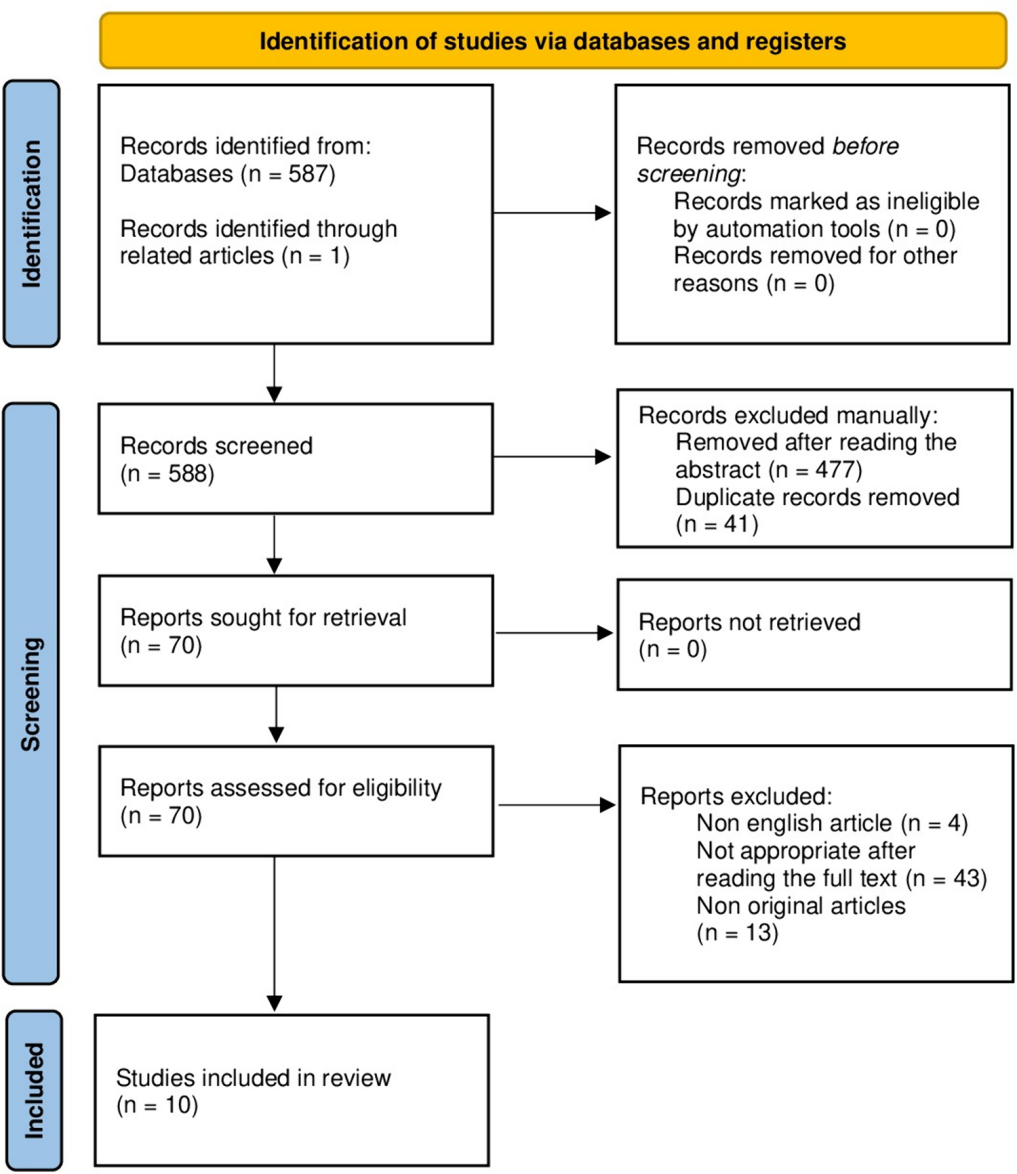
PRISMA flowchart

A PRISMA Flowchart was compiled at the end of the research [Fig. 1].

## Results

A total of 587 articles were found. No articles were removed before screening. 477 articles were excluded after reading the abstract because they were not specific on the early warning system. 41 duplicates were then removed.

69 articles were sought for retrieval and were assessed for eligibility: 4 articles were excluded because full text was not in English; 43 articles were excluded because they were found not appropriate after reading the full text. 13 reviews were excluded because they were not original articles. One article was found and retrieved through related articles.

At the end, a total of 10 articles were included in the review. All studies are published between 2018 and 2024.

We found two retrospectives monocentric, four prospective monocentric, one prospective bicentre, one explanatory sequential mixed method and one monocentric before-after studies.

All studies were performed in Europe, in particular in: Netherlands, Germany, France, UK and Denmark.

Six studies deal with patient who underwent major abdominal surgery, one bariatric surgery; one included traumatology and surgical oncology and another one major non-cardiac surgical procedure in general. The results are summarized in the Results Table [Supplementary Material, Table 1].

### Devices overview

A total of 11 devices were used in the studies included in this review. We will briefly describe the sensor's characteristics, which are all able to perform continuous monitoring. All the devices are CE and/or FDA approved. At the time of writing, the Biosensor BX100® it is not available anymore, replaced by it is successor, the Healthdot®.

Almost all the sensors measured the Heart Rate (HR), except from the Meditech BLUE-BP05® which is blood pressure (BP) only monitor.

Almost all sensors measured the Respiratory Rate (RR), except from Meditech BLUE-BP05® and NoninWristOx 3150®.

The devices capable of monitoring SpO2 are the Masimo Radius-7®, the Biobeat® and the Nonin WristOx 3150®.

2 sensors measured BP: among these, one of them (Biobeat®) is based on a cuffless photopletismografic technology, while the other one (Meditech BLUE-BP05®) it is an oscillometric-based cuff.

Only the Biobeat® was able to measure systolic arterial pressure (SAP), SAP variation, diastolic arterial pressure (DAP) and DAP variation.

HR can be measured both analyzing the waveform, which is the most common way, or by analyzing the electrocardiogram leads like the Isanys Lifetouch®.

The RR may be measured thanks to an accelerometer (Healthdot®), but the way how it is measured it is not clear for all devices.

The most common device design it is a small wireless non-reusable patch applied on patient’s chest. Only one device it is capable of communicating via LoRaWAN technology it is the Healthdot®.

Additionally, many studies were found in the queries on sweat lactate, but none of them concerned the postoperative monitoring so no devices were found.

**Fig. 2:**
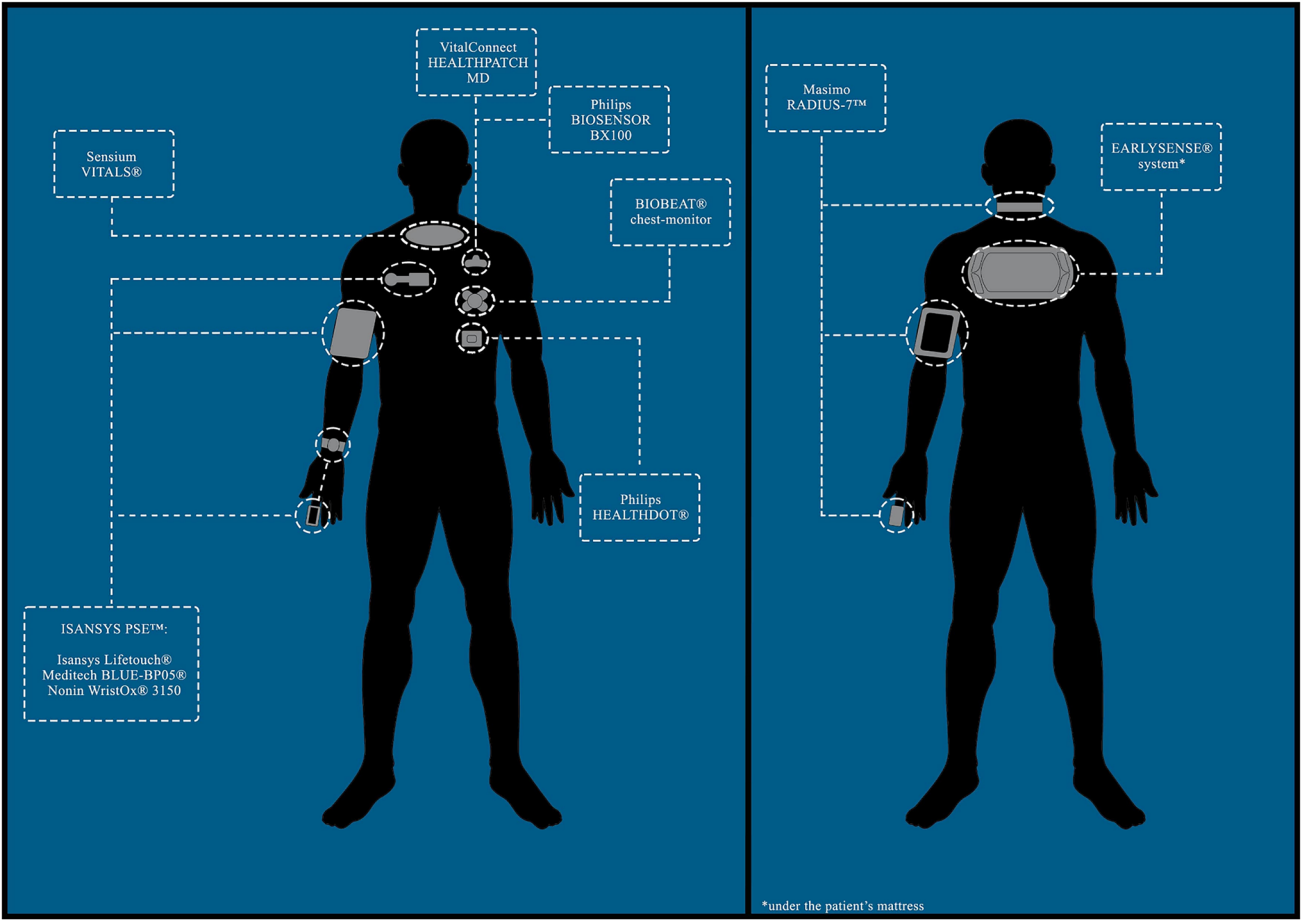
Style of devices and their application

We summarized device’s characteristics described above in [Table 2] and [Fig. 2].

**Table 2:** Devices characteristics

Sensor (manufacturer)	Sensor type	Approval(s)	Vital sign measured
Biobeat chest-monitor (Biobeat Technologies Ltd, Petah Tikva, Israel)	Wireless adhesive patch chest sensor	CE, FDA	HR, RR, SpO2, NIBP, Temperature
Biosensor BX100 (Koninklijke Philips N.V., Amsterdam, Netherlands)	Wireless adhesive patch chest sensor	CE, FDA	HR, RR
EarlySense system (EarlySense Ltd, Ramat Gan, Israel)	Contactless piezoelectric sensor under the patient’s mattress	CE, FDA	HR, RR
IntelliVue® Guardian Solution (Koninklijke Philips N.V., Amsterdam, Netherlands)	Early Warning System (EWS) based on cableless remote controlled devices and monitors connected to a central server	CE, FDA	RR, SpO2, NIBP
Isansys Lifetouch (Isansys Lifecare Ltd, Abingdon, UK)	Wireless adhesive patch chest sensor	CE, FDA*	HR, RR
Healthdot (Koninklijke Philips N.V., Amsterdam, Netherlands)	Wireless adhesive patch chest sensor	CE	HR, RR
HealthPatch MD (VitalConnect, San Jose, CA, USA)	Wireless adhesive patch chest sensor	CE, FDA	HR, RR
Masimo Radius-7 (Masimo Corporation, Irvine, CA, USA)	Patient arm-worn monitor connected to a pulse-oximeter and acoustic adhesive sensor on the neck	CE, FDA	HR, RR, SpO2
Meditech BLUE-BP05 (Meditech Kft., Budapest, Hungary)	Blood pressure monitor	CE, FDA*	NIBP
Nonin WristOx 3150 (Nonin Medical Inc., Plymouth, MN, USA)	Patient wrist-worn monitor connected to a pulse-oximeter	CE. FDA*	HR, SpO2
SensiumVitals (Sensium Healthcare Ltd, Oxford, UK)	Wireless adhesive patch chest sensor	CE, FDA	HR, RR, Skin temperature

*as Isansys Patient Status Engine (PSE)

### Protocols overview

Different protocols were tested. One of them [18] only described a study protocol. Some of them blinded the remote monitoring output, while others set up a reactive or proactive nurse/medical approach to trends or alarms generated by the devices. In all studies the device was applied the day of the surgery. The mean monitoring duration was 6,7 days ranging 72 h to 14 days. However, it is not possible to make a precise estimation in all studies.

**Fig. 3:**
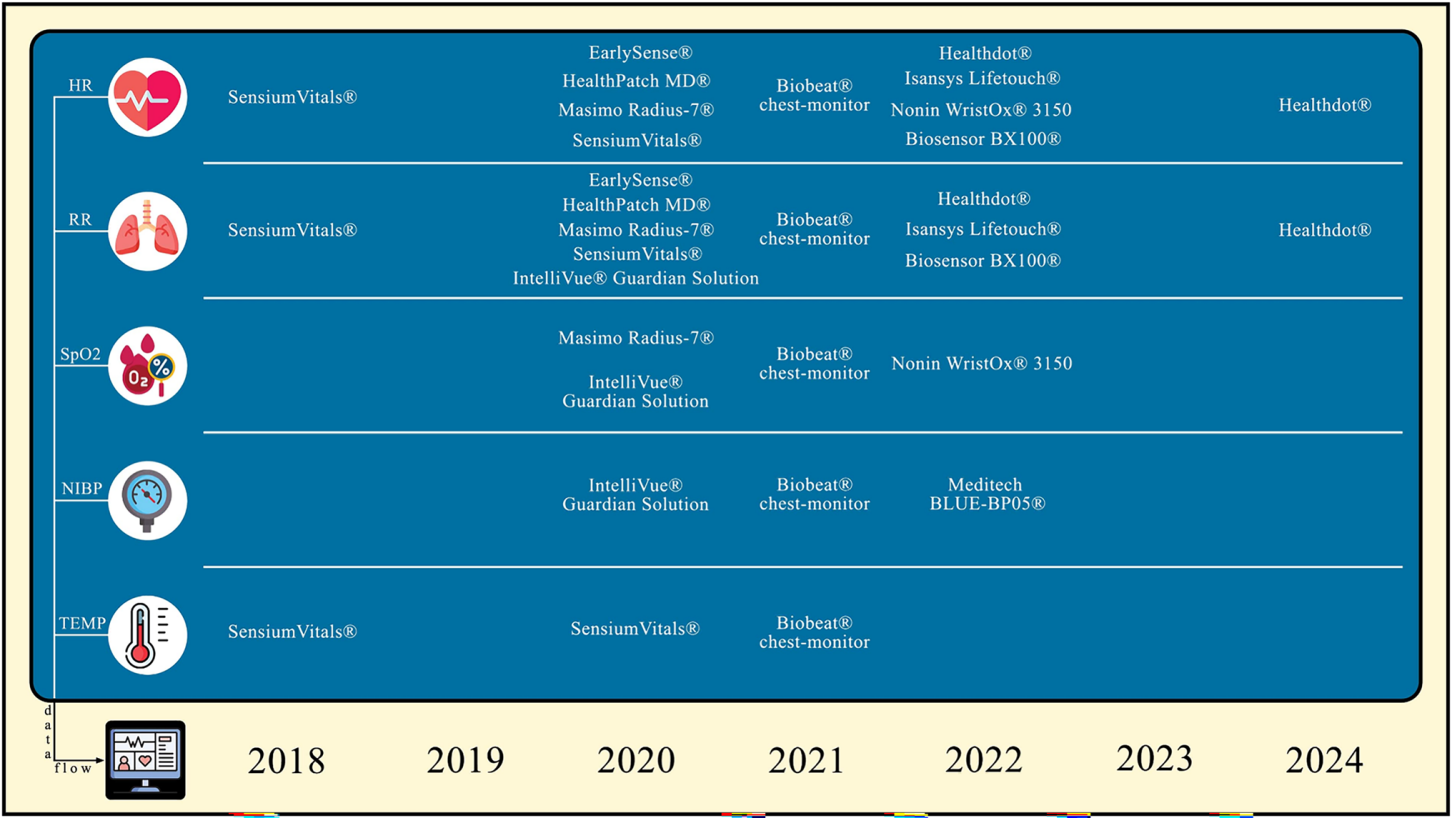
Devices timeline

We will briefly describe them one by one in chronological order [Fig. 3]. Not all Authors extensively described the protocol used, focusing more on their outcome.

Downey C et.al. used a device called SensiumVitals® and described a workflow where nursing staff is involved [19]. The data are transmitted wirelessly every 2 min to a central monitoring station, or a mobile device carried by the patient’s nurse. The nurse is alerted when there is any deviation from preset physiological norms. The alert prompts an acknowledgment of the notification, after which nurses are free to act according to their clinical discretion. Reminders were sent every 14 min until acknowledgment, and levels of engagement were monitored through daily ward visits. This study also demonstrated that the use of these continuous monitoring devices led to faster administration of antibiotics after evidence of sepsis, a shorter average Length of Stay (LOS), and a lower likelihood of readmission within 30 days after hospital stay.

Breteler et.al. compared the trends in the vital signs of patients who developed adverse events with the trends of patients who had not developed any, demonstrating that wireless remote monitoring was able to detect abnormalities in vital signs in patients who presented post-operative complications using 4 devices: SensiumVitals®, HealthPatch®, EarlySense® system and Masimo Radius-7® [8].

In another paper by Downey C, authors randomized patients into an intervention arm that received both a remote monitoring patch and standard NEWS monitoring [20]. Using the SensiumVitals® they monitored heart rate, respiratory rate and temperature continuously. The data ware transmitted wirelessly every 2 min to a mobile device carried by the nurse that alerts when there is deviation from pre-set physiological norms. The patch was activated on arrival to the ward and the patient’s nurse carried a mobile device to alert them if the vital signs strayed outside of normal parameters. Remote monitoring data were also accessible on the ward computer screens for wider access. There was no dedicated telemetry screen for the patch data. Nursing staff were provided with thorough training before the commencement of the study. If the mobile devices alerted the nursing staff to abnormal vital signs, the ensuing clinical response was not mandated but left to the nurse’s discretion within the boundaries of hospital protocols.

Participants in the continuous vital signs monitoring group experienced fewer unplanned critical care admissions (1 vs. 5) and a shorter average hospital stay (11.6 days vs. 16.2 days). The time to receive antibiotics for sepsis was similar in both groups. A cost-utility analysis showed that the remote monitoring system was cost-saving compared to standard NEWS monitoring alone.

Heller AR et.al. [11] installed over two surgical wards 2 spot monitors and 4 sets of wearables cableless devices (integrated with the IntelliVue Guardian Solution®) which sent data to a local area network linked to custom phones where notifications were sent [11]. They do not specify the model of the monitoring system. Cableless devices were used in case of hard workload but not routinely. Spot checks were performed every 2 h, but if a patient had a MEWS 1–4 an alert was sent to nurses; if the MEWS was 5–6 both nurse and physician were alarmed; if MEWS was 7 or more the physician was alerted, and it was up to him if it was needed to activate the emergency team or not. If a life-threatening condition was discovered, with a button on the monitor it was possible to directly alert the emergency team.

The paper by Paternot A et.al. is a bi-centre observational cohort study that included adult patients undergoing non-cardiac surgery hospitalized on an unmonitored general care floor and wearing the Biobeat® chest monitor, a multi-signal wearable sensor, that allows remote monitoring [21]. The study covered the first 72 h after discharge of the patient from the post-anaesthesia care unit. The primary outcome was hemodynamic abnormality. The secondary outcomes were postoperative respiratory and temperature abnormalities, artifacts and blank/null outputs from the wearable device, postoperative complications, and finally, the ease of use of the device. This protocol is specifically aimed to establish whether there is a benefit of remote monitoring using a multiparameter device in the detection of a postoperative complication resulting in an abnormality of one of the major vital signs. However, it is of prime importance to notice that only blood pressure measurement passed validation testing for accuracy and no other parameters measured by the sensor.

Haahr-Raunkjaer C et.al. evaluated the association between abnormal vital signs inspired by Early Warning Score thresholds and subsequent serious adverse events (SAE) in patients undergoing major abdominal surgery and they found no statistically significant association between the total duration of vital sign abnormalities and subsequent occurrence of SAEs [22]. They used 3 different devices: Isansys Lifetouch®, Meditech Blue BP-05® and Nonin WristOx 3150®. However, episodes of tachycardia were seen more often in patients with SAEs, and the number of hypotension episodes was significantly higher in this group, although rare.

Another interesting paper where the aim was to develop a remote early warning system based on continuous measurements using Healthdot® is the one by Van Der Stam J.A. et.al. [15]. They also provided a ground for larger scale follow-up trials that are needed to optimize and validate protocols for the use of wearable based EWS in clinical practice. This study strengthens the idea that remote continuous monitoring with a wearable accelerometer sensor patch has potential as a clinical decision support tool (CDST) while improving patient mobility and comfort as well as workload of nursing staff.

The paper by Van Ede et.al. describes the development of a continuous remote early warning score using the Healthdot® but not how it is implemented in clinical practice. This is an EWS-based notification protocol for deterioration detection in bariatric patients combining thresholds indicative of tachycardia and tachypnea using literature insights and expert sessions [18].

Last but not least, Leenen JP et.al. performed two different studies with different sensors: the Philips Biosensor BX100® and the Healthdot® integrated with the IntelliVue Guardian Solution®. In the first one they highlight the current limitation of the devices given by the presence of unnecessary alarms which would not only lead to discomfort for the patient but also an overload of work for the nursing staff, thus leading to "alarm fatigue" [23]. The purpose of the study was therefore to determine the feasibility of continuous monitoring of vital signs without the use of alarms, thus relying solely on interval trend monitoring. This study shows that standard use of alarms may therefore be reconsidered. In their latest paper they showed how continuous monitoring of vital signs (CMVS) using wearable wireless sensors and proactive trend assessments was associated with a significant decrease in length of stay for colorectal surgery patients but not for hepatopancreatobiliary (HPB) surgery patients [24]. Although all other clinical outcomes were similar in both groups, a non-significant trend towards less-severe complications and reduced ICU LOS was noted in the CMVS group. CMVS with the sensor used in this study was highly accepted by patients. It is important to note that CMVS triggered additional nursing activities such as patient assessments and therapeutic interventions, which may eventually result in attenuation of the severity of postoperative complications.

Wearable devices alone are not sufficient to build a sensor-based continuous early warning system. It is fundamental an interoperable software platform that integrates the data stream coming from the devices. In the articles considered in this review, only the IntelliVue Gurdian Solution® was mentioned as such platform. In addition to this, the IntelliVue Guardian Solution® has a built-in EWS. Nonetheless, it emerges that a well-structured clinical and organizational cascade, along with clear delineation of responsibilities and comprehensive staff training, is essential for effective implementation.

## Discussion

To our knowledge, this is the first review that systematically evaluates the use of wearable devices as part of a continuous remote early warning system.

Although we described how the sensors work, along with their strengths and limitations, our objective was not to evaluate the efficiency or performance of individual devices or to compare them. Instead, we aimed to assess whether these sensors could be useful as part of a CREWS, focusing on the protocol of their application rather than identifying "the best" sensor from a technical perspective.

The deterioration of patient conditions constitutes a burdensome problem in both clinical and financial terms for patients, healthcare professionals, hospitals, and the healthcare system in general [2]. Physicians are increasingly caring for an older patient population affected by multiple pathologies and at higher risk of complications and adverse in-hospital events [3]. As the number of hospital admissions for these patients continues to rise, healthcare professionals and hospitals are being asked to manage a population of patients with more critical health conditions requiring resource-intensive care in an environment where resources are already limited [3]. These factors, combined with the scarcity of beds in more critical departments, mean that patients’ health conditions may be underestimated and that they may be hospitalized or transferred to less critical departments even if they are still at risk of worsening, ending up developing serious adverse events. The clinical, economic, and operational burden of patient deterioration has led to the need for alarm systems that can identify patients at risk and activate intervention based on changes in measurements of key individual physiological parameters.

The standardized EWS systems were created to adopt a multiparametric approach for the identification of small signs of clinical deterioration that precede the occurrence of an event and are made up of two elements: the EWS system to recognize the deterioration, and an emergency team (MET) and/or rapid response (RRT) to manage it adequately. The use of aggregated vital sign scores as used in the MEWS, consisting of 5 parameters, and the NEWS, consisting of 7 parameters, have proven to be more precise than a single parameter system in predicting in-hospital cardiac arrest, mortality, and transfer to intensive care unit within 24 h for adult patients admitted to inpatient departments as demonstrated by the article by Green et.al. [25].

Compared to current clinical practice, the use of wearable devices and the creation of an early, remote, and continuous warning score based on their use has proven to be as safe as MEWS in the early identification of patient clinical deterioration [15]. Indeed, it is advisable to develop CREWS using wearable devices, in fact their use is feasible for clinical practice [18].

It was also found that patients monitored with wearable devices may be less likely to experience an unplanned ICU admission and have a shorter average length of hospital stay if they receive continuous vital signs monitoring compared to those who receive only the usual intermittent monitoring [20]. To confirm this, a study comparing continuous monitoring using wearable devices with intermittent monitoring shows how continuous monitoring is associated with earlier administration of antibiotics in case of sepsis, a reduction in LOS, and how patients are less likely to require readmission within 30 days of discharge [19]. The study by Leenen et al. highlights a reduction in LOS after colorectal surgery in the group of patients monitored with wearable devices compared to that with standard intermittent monitoring. The difference between the two groups, although small, was statistically significant [24].

Although it could be argued that the introduction of these new monitoring devices into clinical practice would require a significant economic investment on the part of departments, the shorter length of hospital stay and the reduction in the number of admissions to intensive care, determined by the use of these devices that guarantee continuous monitoring of patients’ vital parameters, would itself translate into a significant reduction in healthcare costs. All this demonstrates how, despite an initial heavy investment, there would then be savings for hospital companies in terms of patient care costs, while at the same time guaranteeing more careful and timely patient supervision. A further advantage in the introduction of these devices is the reduction of the nursing workload as the healthcare staff would no longer have to manually record the individual parameters patient by patient, but these would be recorded by the device and sent to a monitor for more immediate analysis and overall visualization. All of this would result in more time for nurses to spend on patient care, as well as providing timely warnings of a patient's deterioration, contributing to greater safety. At the same time, however, to avoid the occurrence of unacceptably high false positive alert rates, we highlight how future systems could benefit from improvements in deterioration detection algorithms, leading to real clinical prediction of deterioration and therefore early intervention, as underlined by Breteler et.al. [8]. A possible solution to reduce the incidence of false positives could be the creation of an alarm system that not only takes into account a threshold value to trigger the alarm, as happens for intermittent monitoring, but also the integration with a longitudinal analysis, i.e., the evaluation of the trend of vital signs over time. This trend analysis would enable a more personalized monitoring system based on individual changes in vital signs at predetermined times [22]. In addition to this, a trend analysis could reduce the risk of alarm fatigue, which is linked to reduced compassion and burnout in nurses [23, 26, 27]. One step further: because vital signs alone are not sufficient and nursing assessment is critical to properly activate the emergency team, the introduction of Artificial Intelligence (AI)-based Clinical Decision Support Systems (CDSS) may both support the clinical assessment and reduce the false positives thus reducing the staff workload [27].

This longitudinal analysis of patient parameters would be more appropriate for patients in an inpatient unit; in fact, unlike high care units, patients are not in critical conditions and therefore clinical deterioration tends to be more gradual and acute events are rare, reducing the need for traditional alarm settings for monitoring in general wards. On the other hand, a possible disadvantage of not using threshold alarms is that acute clinical worsening may be detected too late, although this is extremely rare in general wards. This is supported by the study by Leenen et.al., which shows that trend analysis is sufficient for the timely detection of gradual deterioration, whereas alarms would only be useful for the detection of severe acute events, which are extremely uncommon in general wards [23]. Literature indicates that the most indicative parameters of deterioration, particularly cardiac arrest, are tachypnea (58%), tachycardia (54%), altered mental state (46%), arterial hypotension (46%), and poor urine output (29%) [29–31]. However, these are precisely the parameters that are recorded most inconsistently when recorded manually [32].

As stated above, continuous monitoring cannot fully replace nursing care at present, as it lacks the ability to monitor mental state and urine output: nonetheless, it can compensate for the inconsistent recording of some of the most impactful health data, thereby enhancing the reliability of monitoring.

A further advantage of these devices is that they are well tolerated by patients as they are portable, practical, and have little impact on their postoperative hospital activities. In fact, patients considered these devices acceptable in terms of comfort and perceived a greater sense of security [19, 23].

Under the technical point of view, the role of plethysmographic wave-based RR measurement is yet to be defined: the devices which use this technology are not trustable yet because of many artifacts [33, 34]. Anyway, it is interesting that a study that compared the RR measurement of the sensor used by Paternot et al. with a capnographic wave has demonstrated a good precision, even if the underlying technology is photoplethysmography. These results were coherent even after stratification for BMI and skin color [35].

Interestingly, the problems around RR do not concern the loss of data: in one study the HR missing data during time was even greater than RR missing data [36].

Another point of interest is LoRa potential. Only the Heathdot, the device used by Van der Sta et.al., Van Ede et.al., and Leenen et.al. seems to be able to communicate with that flexible and low-cost performing technology [15, 18, 24]. LoRaWAN is a low-cost, long-range, low-economic and energy-cost technology, ideal for applications that require low data rates, such as environmental sensors, smart meters, asset tracking, and remote monitoring [37]. It will probably be the standard for communication for IoT devices. This topic has a great importance because to successfully implement a CREWS in clinical practice, it should be built upon an informatic structure composed by an accessible and interoperable electronic health records (EHRs) and an efficient and secure (privacy by design) real-time data stream. Because the interoperability must be both between EHRs data, between devices and their data output a common integration platform is needed.

The common integration platform needs a standardized data structure, and the semantic interoperability (SI) plays a central role in the standardization. SI in healthcare is crucial for ensuring that different systems can not only exchange data but also accurately interpret and use it. This is achieved using standardized coding systems such as Systematized Nomenclature of Medicine—Clinical Terms (SNOMED CT), International Classification of Diseases (ICD), and Logical Observation Identifiers Names and Codes (LOINC), and frameworks that facilitate consistent communication across various healthcare platforms. These systems ensure that clinical concepts are uniformly represented, allowing different organizations' systems to "speak the same language" and understand data consistently. Uniform language use is essential to avoid ambiguity and enhance understanding among healthcare professionals and information systems. Additionally, frameworks like the European Interoperability Framework (EIF) provide guidelines for implementing semantic interoperability, ensuring clinical data can be reused in different applications without losing meaning.

There are two main standards in healthcare sector: the HL7 FHIR and openEHR.

The HL7 FHIR (Fast Healthcare Interoperability Resources) is designed for the rapid and efficient exchange of clinical data. It uses web technologies like APIs and data formats such as JSON and XML, making integration between different healthcare systems easier. FHIR is known for its ease of implementation and maintenance, making it suitable for applications requiring immediate and flexible access to health information. It also supports AI by providing accessible data models and APIs, facilitating the preparation of data for AI models.

On the other hand, openEHR focuses on the long-term modeling and persistence of clinical data. It is ideal for creating a durable and flexible clinical data repository that can adapt to changes in medical practices and technologies. openEHR emphasizes semantic interoperability by offering a standardized information model and using archetypes to represent complex clinical concepts and their relationships.

The informatic infrastructure, coding systems, and frameworks should be mandated by Health Technology Assessment, particularly when the CREWS relies on AI-processed data. In such cases, an AI Surgical Department should be established in collaboration with engineers and medico-legal professionals, as the delineation of responsibility in this domain remains unclear [38].

Our study has some limitations. First, we included only English articles. Secondly, we assumed a priori that the technology was homogeneous from a technical point of view over the period considered. In addition to this, because we excluded non-adult articles, we cannot generalize the results for the pediatric population. In addition to this, we found a limited number of studies and not all authors described the protocol used extensively. When available, protocols and outcomes where different: we couldn’t perform a rigorous comparative analysis because the studies are methodologically not homogeneous.

The adoption of wearable monitoring devices in healthcare facilities presents numerous challenges that must be carefully considered. One of the main challenges related to the adoption of wearable monitoring devices is the cost. In fact, additional resources are needed to cover the cost of wearable devices which could impact other areas of their budget. As of now, the extent of the reduction of indirect costs like the reduction of LOS of ICU admission is not clear. In addition to this, integrating wearable devices with existing electronic medical record (EMR) systems and other technology infrastructures can be challenging and it may require additional resources, including staff and IT support, to ensure that devices are properly integrated and can be used effectively and safely. The use of these devices would also require an initial adequate training of healthcare workers who need to become more familiar with this new generation of monitors. This may initially increase the workload of the staff, and this could be a barrier to their real use and acceptance. Additionally, wearable monitoring devices collect sensitive health data, which must be protected by privacy regulations. Regular maintenance and calibration are required to ensure the correct operation of the equipment and to improve its reliability and accuracy.

In the evaluation of the various studies, it also emerged that a possible limitation in the applicability of these devices in different geographical areas because of different regulations.

However, our study suggests that healthcare facilities must consider and address the challenges associated with the adoption of these new devices. Adequate budget, integration, training, use permission, data privacy and security, patient acceptance, and device reliability can increase the effectiveness of wearable monitoring devices and improve health outcomes [39].

This paper lays a pragmatic foundation to be used as a solid starting point for the development of new and more extensively described protocols, and for the future application in clinical practice of these monitoring systems.

## Conclusions

We found 10 papers, most of them prospective, which describe and apply real case CREWS protocols using wearable devices and continuous monitoring.

These studies show the potential for these well accepted and convenient devices to be increasingly used in clinical practice as perioperative monitoring systems, also considering the ongoing introduction of devices and software that can be integrated with new technologies such as LoRa and artificial intelligence.

Monitoring vital sign trends, rather than evaluating isolated alarms from wearable devices based on clinician-established thresholds, can enhance decision-making efficiency by fostering a proactive rather than reactive approach.

On the other hand, these promising devices should be validated better under a technical point of view, in particular regarding the RR measurement.

In conclusion, even if further studies are needed to develop and validate existing protocols, the evaluating of vital signs trends with continuous monitoring in non-intensive settings may facilitate the correct and timely identification of postoperative complications and the patient's postoperative setting.

## Supplementary Information

Below is the link to the electronic supplementary material.

Supplementary file1 (DOCX 25 KB)

## Data Availability

No datasets were generated or analysed during the current study.
