# Supplementary material for: Wearable devices as part of postoperative early warning score systems: a scoping review
Source: J Clin Monit Comput. 2024 Oct 8;39(1):233–44. doi: 10.1007/s10877-024-01224-4 (PMC11821718; doi:10.1007/s10877-024-01224-4)
Supplement: Supplementary file 1 — Supplementary file1 (DOCX 25 KB) [file 10877_2024_1224_MOESM1_ESM.docx]

| Authors | Title | Year | Country | Study design | Surgery type | Monitoring setting | Objective | Final cohort | Device Monitoring | Monitoring Duration | Results |
| --- | --- | --- | --- | --- | --- | --- | --- | --- | --- | --- | --- |
| **Downey et al.** | *Continuous Versus Intermittent Vital Signs Monitoring Using a Wearable, Wireless Patch in Patients admitted to Surgical Wards: Pilot Cluster Randomized Trial* | 2018 | UK | Prospective monocentric study  Prospective cluster-randomized, parallel-group. Unblinded, controlled pilot study | Major abdominal surgery | Surgical ward monitoring | The aim was to evaluate whether continuous remote vital signs monitoring is a practical and acceptable way of monitoring surgical patients and to optimize the delivery of a definitive trial | 226 patients | HR  RR  Temperature | At least 5 days | Patients receiving continuous vital signs monitoring had a shorter average length of hospital stay and were less likely to require readmission within 30 days of discharge |
| **Breteler et al.** | *Are current wireless monitoring systems capable of detecting adverse events in high-risk surgical patients? A descriptive study* | 2020 | Netherlands | Prospective observational monocentric study | Traumatology, surgical oncology | Step-down Unit | Describe the ability of currently available sensors to detect vital signs changes prior to and during these events in a group of high-risk surgical patients. | 31 patients | HR  RR  SpO2  (no alarms were generated and sent to nurses) | Median 88h/patient | Wireless patient monitoring systems on the ward are capable of detecting  abnormalities in vital sign patterns in patients who develop adverse events. Remote patient monitoring  may have potential to improve patient safety by generating early warnings for deterioration to nursing  staff.  Twenty adverse events occurred in 11 patients, of which 9 (45%) during SDU stay and 11 (55%) at the surgical ward. |
| **Downey et al.** | *Trial of remote continuous versus intermittent NEWS monitoring after major surgery (TRaCINg): a feasibility randomized controlled trial* | 2020 | UK | Prospective monocentric study  Monocentric randomized, controlled, unblinded, parallel group, feasibility trial | Elective major abdominal surgery | Surgical ward monitoring | The main aim of the study was to determine the feasibility of performing a large-scale individually randomized controlled trial of continuous remote monitoring after major surgery.  Informally assess the potential safety, potential efficacy, acceptability and potential cost utility of a wearable, remote monitoring system for patients after major surgery, as compared to standard monitoring with the NEWS system alone. | 136 patients | HR  RR  Temperature  (data transmitted to nurses) | Entire hospital stay or at least 5 days during admission | The cost-utility analysis indicated that the SensiumVitals remote monitoring system had the potential to be cost-saving when compared to standard NEWS monitoring alone |
| **Heller A.R. et al.** | *Detection of Deteriorating Patients on Surgical Wards Outside the ICU by an Automated MEWS-Based Early Warning System With Paging Functionality* | 2020 | Germany | Retrospective, monocentric case-control study | Complex surgical interventions | Surgical ward | Evaluate the effect of deploying an automated MEWS-based EWS with telemetry | Control cohort: 1896 patients  Intervention cohort: 1931 patients | RR  NIBP  SpO2 | Surgical ward staying | In concert with a well trained staff, the early deterioration detection of patients in surgical ward may be improved by the introduction of an automated MEWS-based EWS |
| **Paternot et al.** | *Screening for postoperative vital signs abnormalities, and particularly hemodynamic ones, by continuous monitoring: protocol for the Biobeat-Postop cohort study* | 2021 | France | Prospective observational bi-center study | Major non-cardiac surgical procedure (gastro-intestinal, gynaecological, urologic, orthopaedic) | Post PACU discharge | Study protocol is specifically aimed to establish whether there is a benefit of remote monitoring using a multiparameter device in the detection of a postoperative complication resulting in an abnormality of one of the major vital signs. The main outcome of the study which is the occurrence of one or more episodes with a mean arterial pressure below the threshold of 60 mmHg during the first 72 postoperative hours. | 114 patients | HR  RR  SpO2  NIBP (SAP, DAP, and variations)  Temperature  (staff do not have access to data) | 72h | Ongoing study |
| **Haahr-Raunkjaer et al.** | *Continuous monitoring of vital sign abnormalities: association to clinical complications in 500 postoperative patients* | 2022 | Denmark | Prospective monocentric study  Prospective observational cohort study | Major abdominal surgery | Surgical ward monitoring | The objective of the study was to evaluate the association between abnormal vital signs inspired by Early Warning Score thresholds and subsequent SAEs (serious adverse events) in patients undergoing major abdominal surgery | 491 patients | HR  RR  SpO2  NIBP  (data blinded to staff) | Up to 96h after surgery | Overall duration of vital sign abnormalities at current thresholds were not significantly associated with subsequent serious adverse events, but more patients with tachycardia and hypotension had subsequent serious adverse events.  Continuous wireless monitoring is feasible, including 92% of the potential observation time with at least one device measuring. Standard blood pressure monitors were not continuously measuring (measurements every 30 or 60 min), and with the used devices, it would not be possible to measure continuously due to patient discomfort |
| **Van der Stam et al.** | *A wearable patch based remote early warning score (REWS) in major abdominal cancer surgery patients* | 2022 | Netherlands | Prospective monocentric study | Major abdominal cancer surgery | Post PACU discharge monitoring | Development of a remote early warning score (REWS) using vital parameters collected by a wearable sensor and compare its diagnostic performance for the detection of deterioration to the diagnostic performance of the conventional modified early warning score (MEWS). | 103 patients | HR  RR  (staff did not have access to data) | 14 days | A remote early warning scoring system based on vital parameters measured by a wearable patch offers a predictive performance comparable to that of the MEWS in patients following major abdominal cancer surgery. High sensitivity and specificity. |
| **Van Ede et al.** | *Continuous remote monitoring in post-bariatric surgery patients: development of an early warning protocol* | 2022 | Netherlands | Retrospective monocentric study  Internally validated protocol | Bariatric surgery  (laparoscopic sleeve gastrectomy or laparoscopic Roux-en-Y gastric bypass) | Surgical ward | Development EWS-based notification protocol for deterioration detection in bariatric patients.  Describe the process of the development of a continuous remote early warning score (CREWS) protocol. | 184 patients  Additional home data from 162 patients were available | HR  RR | 14 days postoperatively | Most protocols resulted in an overall sensitivity of 75%: 100% in hospital and 66% at home.  Implementation of a protocol with a threshold of 110 bpm for heart rate and 20 rpm for RR may be feasible, while the use of beta-blockers seemed not to necessitate a protocol setting. |
| **Leenen et al.** | *Feasibility of wireless continuous monitoring of vital signs without using alarms on a general surgical ward: a mixed methods study* | 2022 | Netherlands | Explanatory sequential mixed methods study | Abdominal surgery | Surgical ward | The aim of this study was to determine the feasibility of continuous vital sign monitoring without the use of alarms, thereby exclusively relying on interval trend monitoring.  Instead of using alarms we focused exclusively on regular vital sign trend analysis by nurses and doctors | 56 patients | HR  RR | At least 5 days postoperatively at ward since the arrive of the patient from ICU or recovery | They demonstrated that continuous vital signs trend monitoring without using alarms was feasible in the general ward setting, thereby avoiding unnecessary alarms and preventing alarm fatigue. When monitoring in a general ward setting, the standard use of alarms may therefore be reconsidered |
| **Leenen et al.** | *Impact of wearable wireless continuous vital sign monitoring in abdominal surgical patients: before-after study* | 2024 | Netherlands | Monocentric before-after study | Major abdominal surgery | The primary aim was to explore the effect of continuous monitoring of vital signs (CMVS) on length of hospital stay (LOS). Secondary aims were to explore the effects of CMVS on a broad range of other clinical outcome measures | The primary aim was to explore the effect of continuous monitoring of vital signs (CMVS) on length of hospital stay (LOS). Secondary aims were to explore the effects of CMVS on a broad range of other clinical outcome measures | 908 patients | HR  RR | Entire hospital stay | Even though the observed reduction in LOS may suggest that CMVS has enabled more rapid detection and intervention in case of clinical deterioration, no significant differences were found in complication rate, complication severity, RRT calls, ICU admissions and ICU LOS |

Tab.2: Results Table
